# Interaction between nuclear‐translocated cellular communication network factor 2 and purine‐rich box 1 regulates the expression of fibrosis‐related genes

**DOI:** 10.1002/ccs3.70051

**Published:** 2025-09-25

**Authors:** Xuan Thi Nguyen, Satoshi Kubota, Masaharu Takigawa, Takashi Nishida

**Affiliations:** ^1^ Department of Biochemistry and Molecular Dentistry Okayama University Graduate School of Medicine, Dentistry and Pharmaceutical Sciences Okayama Japan; ^2^ Advanced Research Center for Oral and Craniofacial Sciences Okayama University Faculty of Medicine, Dentistry and Pharmaceutical Sciences Okayama Japan

**Keywords:** cellular communication network factor 2 (CCN2), fibrosis, myofibroblast, purine‐rich box 1 (PU.1), transcription co‐factor

## Abstract

Cellular communication network factor 2 (CCN2) with a nuclear localization signal‐like peptide is known to promote fibrosis. However, translocation of CCN2 into the nucleus and its role in fibrosis remain unclear. We hypothesized that nuclear‐translocated CCN2 is associated with purine‐rich box 1 (PU.1), which is a transcription factor regulating the differentiation of myofibroblasts. Western blot analysis of the cytoplasmic and nuclear fractions of cell lysate and immunofluorescence analysis revealed that CCN2 was detectable in both the cytoplasm and nuclei of murine fibroblastic NIH3T3 cells. Additionally, chromatin immunoprecipitation (IP)‐PCR and an electrophoretic mobility shift assay revealed that recombinant CCN2 protein bound to the regulatory region of *Spi1*, which encodes PU.1. Furthermore, IP‐Western blot analysis showed that CCN2 interacted with PU.1. Finally, the forced expression of both *Ccn2* and *Spi1* significantly promoted the production of angiotensin II, and increased fibrosis‐related molecules, such as *Col1a1* and *Acta2*, at the gene and protein levels. These findings indicate that CCN2 translocated to the nucleus interacts with PU.1 and that the complex promotes the markers of myofibroblast differentiation, suggesting that CCN2 plays an important role in fibrosis via cooperation with PU.1, as a transcription co‐factor.

## Graphical abstract

Although cellular communication network factor 2 (CCN2) is secreted out to the extracellular environment, intracellular CCN2 rather than re‐incorporated CCN2 from the outside may be involved in its translocation into the nuclei, because translocation of CCN2 into the nucleus was not disturbed by soluble heparin. Nuclear‐localized CCN2 interacts with the *Spi1* promoter and increases PU.1 production. Furthermore, the complex of PU.1 and CCN2 increases fibrosis‐related factors, such as type I collagen and α‐smooth muscle actin.

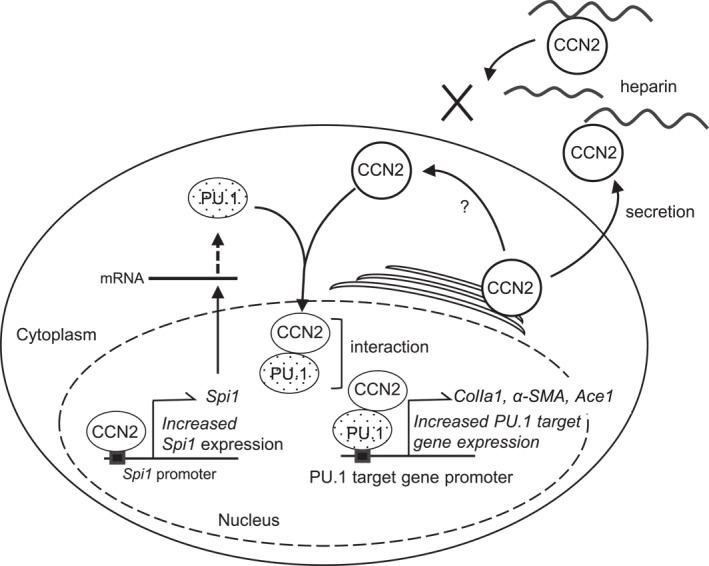

## 1 INTRODUCTION

Fibrosis is a disease in which excessive fibrous connective tissue accumulates in organs when the wound healing of injured organs and tissues is uncontrolled.^1^, ^2^ As a result, the organs and tissues lose their flexibility, and their normal functions are impaired.^1^, ^2^ In fact, fibrosis in patients suffering from various diseases has been identified to have a close relationship with disability and death.^3^ Under such pathological conditions, it has been suggested that fibroblasts transdifferentiate to myofibroblasts, which excessively produce components of connective tissue, such as type I collagen and α‐smooth muscle actin (α‐SMA).^4^ However, the mechanism that regulates this trans‐differentiation remains largely unclear.

Purine‐rich box 1 (PU.1) was originally known as a transcription factor that promotes the differentiation from CD34‐positive hematopoietic stem cells to B lymphocytes and inhibits differentiation to pre‐erythroblasts,^5^ but it was recently reported that PU.1 is also a critical regulator of pro‐fibrotic system; this was stated on the grounds that fibrosis did not occur in *Spi1* knocked‐out mice encoding PU.1 and that *Spi1*‐overexpressed fibroblasts promoted differentiation to myofibroblasts.^6^ On the other hand, cellular communication network factor 2 (CCN2) has also been reported to be related to the onset and progression of fibrosis.^7^, ^8^ CCN family proteins, including CCN2, are matricellular proteins that mainly act in an extracellular microenvironment and are composed of six proteins, named CCN1 to CCN6.^9^, ^10^ It has been reported that the conservation degree of the nucleotide sequences is approximately 30%–50%, and that the similarity in amino acid sequences is approximately 40%–60%.^11^ However, the tetra‐modular structure among the family members is well‐conserved except for CCN5, which lacks the fourth module, namely a carboxyl terminal cystine knot.^9^, ^10^ These proteins coordinate various cellular functions, such as extracellular matrix (ECM) remodeling, skeletal development, angiogenesis, and wound healing under physiological conditions. Conversely, it has been reported that, under pathological conditions, these factors are involved in inflammation and tumor development.^9^, ^10^ In particular, in terms of fibrosis induced in various diseases, the promotion or suppression of fibrosis by CCN family proteins has been reported.^7^ For example, CCN1 mediates cell adhesion,^12^ apoptosis,^13^ and the cell senescence of fibroblasts by binding to integrins and heparan sulfate proteoglycans.^14^ Moreover, it has been reported that CCN1 is expressed in myofibroblasts and mediates cell senescence,^15^ thus suggesting that CCN1 has a suppressive effect on fibrosis. Similarly to CCN1, CCN3 is known as a negative regulator of ECM accumulation.^16^ It was reported that the gene expression levels of *Acta2*, which encodes α‐SMA, and *Col1a1*, which are markers of myofibroblasts, were downregulated in murine fibroblasts with transiently overexpressed exogenous *CCN3*.^17^ Additionally, CCN5 has also been reported to suppress fibrosis^18^; however, the effect of CCN6 on fibrosis is still controversial because it has been reported that CCN6 either promotes or suppresses fibrosis.^19^, ^20^ In contrast to the effects of CCN1, CCN3, and CCN5 on fibrosis, the promotive effects of CCN2 and CCN4 on fibrosis have been clearly demonstrated.^8^, ^21^, ^22^ In particular, CCN2 has been reported as a pivotal regulator that aggravates fibrosis since its discovery. Consequently, FG‐3019 (Pamrevlumab), which is a humanized anti‐CCN2 monoclonal antibody, was used in clinical trials for the treatment of idiopathic pulmonary fibrosis (IPF) in the USA.^23^ Therefore, to understand the pathological conditions of fibrosis, it is very important to clarify the role of CCN2 in detail.

CCN2 has not only an N‐terminal signal peptide, demonstrating its function as a secretory protein, but also a nuclear localization signal‐like (NLS‐like) peptide, which is enriched in lysine and arginine residues around the C‐terminus.^24^ Indeed, it was previously reported that CCN2 was located in the cytoplasm and translocated into the nucleus in mesangial and melanoma cells.^24^, ^25^ However, the role of CCN2 translocated into the nucleus remains unclear. In general, a factor that has both an effect via receptors on the cell membrane and an intracellular effect is called an intracrine factor.^26^ CCN2 that has both signal peptide and NLS‐like peptide may be an intracrine factor. So far, the roles of secreted CCN2 in fibrosis have been investigated, but the role of nuclear‐translocated CCN2 has been largely remained unknown. Recently, the results of phase III randomized clinical trial using FG‐3019 for the treatment of IPF was reported.^27^ Contrary to expectations, the effect of FG‐3019 on the treatment of IPF was not observed.^27^ Considering the outcomes observed in phase III trials, intracellular CCN2, particularly CCN2 translocated into the nucleus may be more involved in the progression of fibrosis than CCN2 secreted into an extracellular microenvironment.

The aim of this study is to clarify whether or not CCN2 is translocated into the nucleus of fibroblasts, and also to investigate whether or not CCN2 translocated into the nucleus plays a role in the expressions and productions of fibrotic markers via cooperation with PU.1.

## 2 MATERIALS AND METHODS

### 2.1 Materials

Cell culture dishes and multi‐well plates were purchased from Corning and Thermo Scientific. Dulbecco's modified Eagle's medium (DMEM) and fetal bovine serum (FBS) were purchased from Shimadzu Diagnostics Co. Ltd. and Nichirei Biosciences Inc., respectively. A list of antibodies used in this study is provided in Table S1. Soluble heparin and losartan potassium salt were purchased from Tokyo Chemical Industry Co. Ltd. and Fujifilm Wako Pure Chemical, respectively. Recombinant CCN2 protein (rCCN2) was purified as described previously.^28^

### 2.2 Cell cultures

Murine embryonic skin fibroblastic NIH3T3 cells were inoculated at a density of 1.5 × 10^4^ cells/cm^2^ into culture dishes and multi‐well plates containing DMEM supplemented with 10% FBS and cultured at 37°C in a humidified atmosphere with 5% CO_2_.

### 2.3 Plasmid constructs

A pFlag‐CMV (Invitrogen)‐derived *CCN2*‐HA plasmid (p*CCN2*‐HA), in which the full‐length coding region of mouse *CCN2* bearing an HA‐tag at the C‐terminal region was inserted, was kindly provided by Dr. T. Hattori. Additionally, pFlag‐*Spi1*, the full‐length coding region of *Spi1*, which encodes PU.1, was amplified via polymerase chain reaction (PCR) using cDNA derived from a murine macrophage cell line as a template, and then inserted into the pFlag‐CMV plasmid. Moreover, glutathione‐S‐transferase (GST) with CCN2's NLS‐like peptide (pFlag‐GST‐NLS) and GST only (pFlag‐GST) were inserted into pFlag‐CMV plasmid, respectively. The nucleotide sequences of all the plasmids were confirmed using Sanger sequencing. These cDNAs were generated using the gene‐specific primers listed in Table S2A.

### 2.4 Gene transfection

After being treated with 0.25% trypsin, NIH3T3 cells were collected; then, 1.0 × 10^6^ cells were re‐suspended in OPTI‐MEM (Fisher Scientific) containing 10 or 20 μg of plasmids. Electroporation was performed using an NEPA21 (NEPA GENE Co., Ltd.) according to the previous gene transfer condition^29^ and the manufacturer's instructions. Thereafter, the cells were cultured for 48 h for subsequent experiments.

### 2.5 Indirect immunofluorescence analysis

NIH3T3 cells were transfected with p*CCN2*‐HA and/or pFlag‐*Spi1,* and were cultured in four‐well glass slide chambers (Nalge Nunc International) for 48 h. These cells were then fixed with 4% paraformaldehyde for 1 h at room temperature and made permeable with 0.1% NP‐40 in phosphate‐buffered saline (PBS) for 10 min. Indirect immunofluorescence analysis was performed as described previously.^30^ Briefly, the permeabilized cells were reacted with anti‐Flag, anti‐HA, anti‐CCN2, anti‐PU.1, and/or anti‐GST antibodies (Table S1) in PBS containing 3% bovine serum albumin (BSA) overnight at 4°C. After being washed with PBS three times, the samples were incubated with secondary antibodies (Alexa Fluor 568 goat anti‐mouse, Alexa Fluor 568 goat anti‐rabbit, Alexa Fluor 488 goat anti‐mouse, and anti‐goat IgG TRITC conjugate) for 1 h at room temperature. After being washed with PBS three times, immunoreactivity was observed with a fluorescence microscopy (FSX100, Olympus).

### 2.6 Quantitative reverse transcription‐PCR analysis

Total RNAs from NIH3T3 cells were isolated using ISOGEN reagent (Nippon Gene). First‐strand cDNA was synthesized from 1 μg of total RNA using a PrimerScript^TM^ RT reagent kit (Takara Shuzo), and subsequent quantitative PCR analysis was performed with a SYBR^®^ green real‐time PCR master mix (Toyobo) and specific primers using a StepOne plus real‐time PCR machine (Applied Biosystems), as described previously.^31^ Glyceraldehyde 3‐phosphate dehydrogenase gene (*Gapdh*) was used as a reference, and the gene expression levels were evaluated using the 2^−ΔΔCt^ method.^32^ The specific primer sequences and the accession numbers of the target mRNAs are shown in Table S2B.

### 2.7 Western blot analysis

NIH3T3 cells were transfected with p*CCN2*‐HA, pFlag‐*Spi1*, pFlag‐GST, or pFlag‐GST‐NLS. After 48 h, the nuclear and cytoplasmic proteins were separated using a PARIS^TM^ kit (Life Technology) according to the manufacturer's protocol. The nuclear extracts were sonicated on ice and centrifuged at 10,000 g for 5 min to remove debris. Next, the protein concentrations of the nuclear and cytoplasmic proteins were determined using a Pierce^TM^ BCA protein assay kit (Thermo Fisher Scientific) with serially diluted BSA as a standard. A Western blot analysis was performed as described previously.^33^ Briefly, the extracts were separated using sodium dodecyl sulfate polyacrylamide gel electrophoresis and were then transferred to polyvinylidene difluoride membranes (Millipore‐Sigma) using a semi‐dry transfer apparatus (Atto Corp.). Blots were then reacted overnight at 4°C with primary antibodies used at an indicated dilution in Table S1. Then, after being washed with Tris‐buffered saline containing Tween 20 (TBST) and TBS buffers, the blots were reacted for 1 h at room temperature with secondary antibodies conjugated with horseradish peroxidase. Subsequently, the membranes were washed for 15 and 10 min with TBST and TBS buffers, respectively, and the bands were detected with a chemiluminescence substrate by using a LAS‐4000 mini image analyzer (Fuji Film).

### 2.8 ChIP assay

The chromatin immunoprecipitation (ChIP) assay was performed according to the protocol used in a previous report with slight modifications.^34^ Briefly, the plasmid‐transfected cells were collected using 0.25% trypsin and washed with a fresh medium containing 10% FBS. The cells were fixed using 1% paraformaldehyde and lysed in SDS lysis buffer (50 mM Tris‐HCl pH 8.0, 10 mM EDTA, 1% SDS) containing 1% protease inhibitor cocktail (Sigma Aldrich) for 20 min on ice. To cleave the genome DNA, 15 s intervals of sonication were performed 10 times on ice. After centrifugation at 16,000 g at 4°C to remove debris, the supernatant was diluted 10‐fold in ChIP dilution buffer (50 mM Tris‐HCl pH 8.0, 167 mM NaCl, 1% Triton X‐100%, and 0.1% sodium deoxycholate) with 1% protease inhibitor cocktail (Sigma Aldrich), and 10% of this sample was retained as the input sample. The remaining sample was reacted with anti‐CCN2 antibody while rotating overnight at 4°C using a rotator and was then mixed with Protein G‐Sepharose (GE Healthcare Bio) at room temperature for 2 h to pull down the immune complex. Non‐immune control rabbit IgG was used as the negative control. The cell lysate‐antibody mixture and input samples were treated with RNase A for 30 min at 37°C and then treated with proteinase K (10 mg/mL) for 1 h at 55°C. The samples were extracted with phenol‐chloroform, and DNA was recollected using ethanol precipitation in the presence of 40 μg of glycogen and resuspended with 20 μL of Tris‐EDTA buffer. After the measurement of the DNA concentration, these samples were used for PCR analysis.

### 2.9 PCR analysis

For PCR analysis, a 10 μL reaction mixture containing 0.2 μM of each primer, 100 ng of ChIP DNA, and 2 × Quick Taq HS Dye Mix (Toyobo) was subjected to 35 cycles of PCR at 94°C for 30 s, 60°C for 30 s, and 68°C for 30 s followed by 10 min at 68°C. A total of 10 μL of the PCR products were run on 1% agarose gels and visualized with ethidium bromide staining. The specific primer sequences are shown in Table S2C.

### 2.10 Electrophoretic mobility shift assay

Electrophoretic mobility shift assay (EMSA) was performed according to a previous report.^34^ Briefly, to prepare the probe for the *Spi1* promoter region, PCR was performed using the genomic DNA of the NIH3T3 cells as a template, with biotin‐labeled specific primers made based on the *Spi1* promoter proximal region that is conserved among various species, as shown in Table S2D. The PCR products were cut out from the agarose gel and purified. Next, for the interaction between CCN2 and the probe, the biotin‐labeled probes and rCCN2 or BSA were added to 20 mM HEPES, 45 mM KCl, 10 mM NaCl, 1 mM dithiothreitol, 0.1% NP‐40%, and 10% glycerol and were kept at 4°C overnight. The reaction mixture was separated in 4% polyacrylamide gels in 0.5% Tris‐boric acid‐EDTA buffer, and then transferred to positively charged nylon membranes (Roche) using a wet tank transfer apparatus (Bio‐Rad). After UV cross‐linking, the membrane was soaked with 5% skim milk for 30 min and incubated with an HRP‐labeled streptavidin diluted 2000 times for 1 h at room temperature. After being washed 5 times with TBST and 3 times with TBS for 5 min, the biotinylated bands were detected with a chemiluminescence substrate using a LAS‐4000mini image analyzer (Fuji Film).

### 2.11 IP‐Western blot analysis

Immunoprecipitation (IP)–Western blot analysis was performed as described previously.^30^ After the NIH3T3 cells were transfected with p*CCN2*‐HA and pFlag‐*Spi1*, the nuclear extract was prepared using a PARIS kit and reacted with an anti‐PU.1 antibody or non‐immune control mouse IgG at a concentration of 10 μg/mL at 4°C overnight. Protein G‐Sepharose (GE Healthcare Bio) was then added and mixed by swirling at room temperature for 2 h. After being washed with PBS 5 times for 5 min, the bound proteins were extracted using an SDS sample buffer with 2‐mercaptoethanol. Western blot analysis was performed using an anti‐HA or anti‐PU.1 antibody.

### 2.12 Enzyme‐linked immunosorbent assay (ELISA) for ANG II

NIH3T3 cells were transfected with p*CCN2*‐HA and pFlag‐*Spi1,* or an empty vector (EV). After 48 h, the cell lysate was harvested, and angiotensin II (ANG II) concentrations were determined using a commercial ELISA kit (Enzo Life Sciences), following the manufacturer's instructions. Samples from 4 independent experiments were analyzed, and the mean and standard deviation (SD) were calculated using Boster Bio's ELISA data assay tool (https://www.bosterbio.com/biology‐research‐tools/elisa‐data‐analysis‐online?).

### 2.13 Statistical analysis

All the experiments were repeated at least twice, and similar results were obtained. After performing an *F*‐test (testing for normality), Bonferroni's test and an unpaired Student's *t*‐test were performed to compare the data from multiple groups and from two groups, respectively. All data are shown as the mean values and SDs.

## 3 RESULTS

### 3.1 CCN2 is translocated into the nucleus in NIH3T3 cells

**FIGURE 1:**
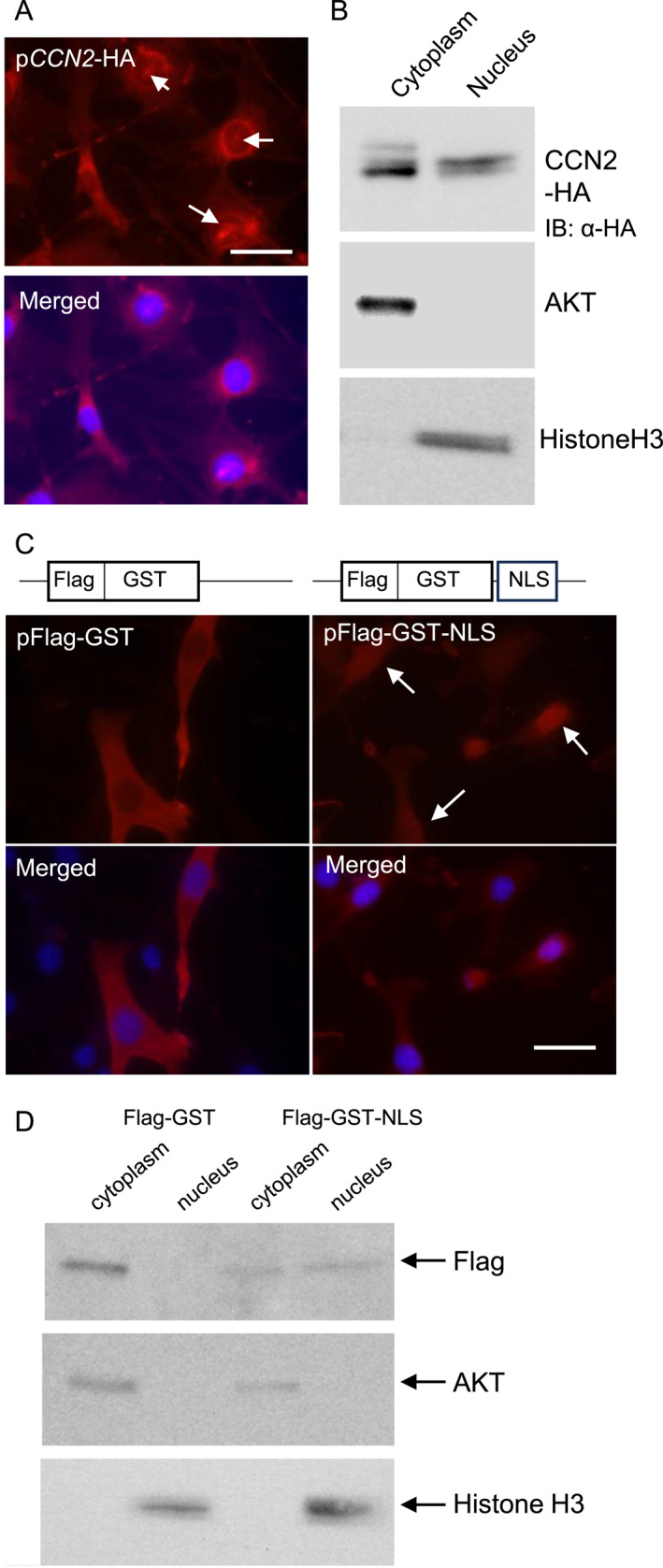
Translocation of CCN2 into the nucleus in fibroblasts. (A) Indirect immunofluorescence analysis using anti‐HA antibody. After the NIH3T3 cells were transfected with p*CCN2*‐HA, they were cultured in four‐well glass slide chambers for 48 h. An indirect immunofluorescence analysis was then performed using an anti‐HA antibody. The arrows indicate CCN2 translocated into the nucleus. DAPI staining was performed in the same fields. The bar represents 30 μm. (B) Western blot analysis of CCN2 in the cytoplasmic and nuclear cell lysate of NIH3T3 cells. After the NIH3T3 cells were transfected with p*CCN2*‐HA and cultured for 48 h, the cell lysate was separated into cytoplasmic and nuclear fractions. A Western blot analysis was then performed. AKT and Histone H3 are markers of the cytoplasm and nucleus, respectively. (C) Indirect immunofluorescence analysis using anti‐GST antibody. After the NIH3T3 cells were transfected with pFlag‐GST or pFlag‐GST‐NLS, the cells were cultured in four‐well glass slide chambers for 48 h. An indirect immunofluorescence analysis was then performed with an anti‐GST antibody. The arrow indicates GST translocated into the nuclei. DAPI staining was merged in the same fields. The bars represent 30 μm. (D) Western blot analysis of Flag‐conjugated GST in the cytoplasmic and nuclear cell lysate of NIH3T3 cells. NIH3T3 cells were transfected with pFlag‐GST or pFlag‐GST‐NLS and then cultured for 48 h. A Western blot analysis was performed using an anti‐Flag antibody. AKT and Histone H3 are markers of the cytoplasm and nucleus, respectively. Positions of the molecular weight markers are shown at the left. CCN2, cellular communication network factor 2 and GST, glutathione‐S‐transferase.

To investigate whether CCN2 is translocated into the nucleus or not, we first performed an immunofluorescence analysis in the NIH3T3 cells transfected with p*CCN2*‐HA. As shown in Figure 1A, CCN2 detected with the anti‐HA antibody was partially located in the nuclei (see the arrows). To support this finding, a Western blot analysis was carried out using p*CCN2*‐HA‐transfected NIH3T3 cell lysate separated into nuclear and cytoplasmic fractions. AKT and Histone H3 were used as markers of the cytoplasm and nucleus, respectively. As shown in Figure 1B, the bands of immunoreactivity for HA were detected in both the cytoplasmic and nuclear fractions of the cell lysate. These findings suggest that CCN2 can be translocated into the nucleus. Next, to investigate whether or not the NLS‐like peptide that exists in CCN2 is involved in CCN2's translocation into the nucleus, we constructed two plasmids; one expresses GST containing the NLS‐like peptide (pFlag‐GST‐NLS), and the other expresses GST only (pFlag‐GST). As shown in Figure 1C, the immunofluorescence analysis revealed that the signals for Flag‐conjugated GST containing the NLS‐like peptide were detected in both the nucleus and cytoplasm of NIH3T3 cells (see the arrows), and the signals for Flag‐conjugated GST were detected in the cytoplasm only. Furthermore, the results of the Western blot analysis in Figure 1D were shown to be similar to the results of Figure 1C. Namely, immunoreactivity for Flag‐conjugated GST was detected in the nucleus and cytoplasm of NIH3T3 cells transfected with pFlag‐GST‐NLS, whereas it was only detected in the cytoplasm of the cells transfected with pFlag‐GST. These findings indicate that the NLS‐like peptide localized in CCN2 is capable of translocating proteins to the nucleus in *cis*.

### 3.2 CCN2's translocation into the nucleus upregulates Spi1 expression

**FIGURE 2:**
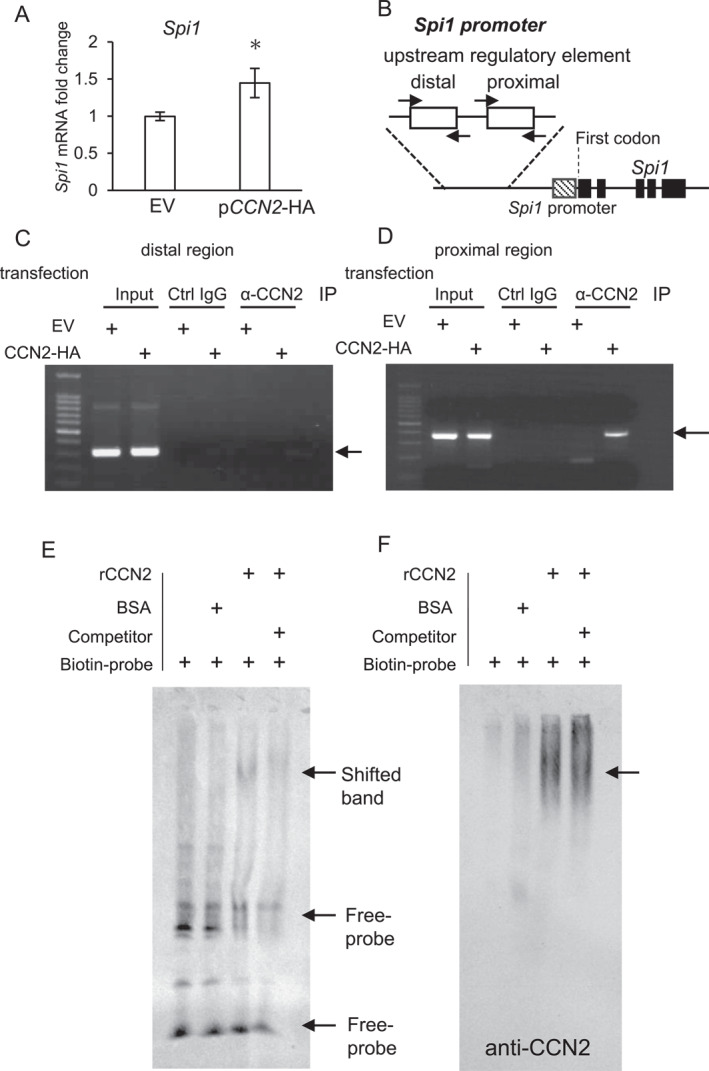
Gene expression of PU.1 under the forced expression of CCN2 and its interaction with the regulatory element of the *Spi1* expression. (A) *Spi1* expression level in NIH3T3 cells transfected with p*CCN2*‐HA. After 48 h, the total RNAs were isolated, and a quantitative RT‐PCR analysis was performed. The amount of *Gapdh* mRNA was used as a reference. Delta–delta Ct (ΔΔCt) values were used for the relative quantification of these gene expressions. The bars represent mean and standard deviation data from independent culture dishes (*n* = 4) and indicate the relative ratio with respect to the transfection with EV (EV = 1.0). The data was analyzed using Student's *t*‐test. The asterisk indicates a significant difference from the transfection with EV at *p* < 0.05. (B) Schema of ChIP‐PCR primer binding sites in the upstream regulatory elements of *Spi1* gene. The black, striped, and white boxes indicate the exon of the *Spi1* gene, *Spi1* promoter, and the PCR products amplified with specific primers (arrows), respectively. (C, D) Detection of the proximal regulatory region binding to CCN2, utilizing the ChIP‐PCR technique. NIH3T3 cells transfected with p*CCN2*‐HA were cultured for 48 h, and the cell lysate was collected. After sonication, the input samples were aliquoted, and immunoprecipitation was performed using an anti‐CCN2 antibody. Genomic DNA was purified using the phenol‐chloroform method, and PCR analysis was carried out by using specific primers for distal (C) and proximal binding sites (D) in the *Spi1* regulatory region. The PCR products were then electrophoresed in a 1% agarose gel and visualized with ethidium bromide staining. The arrows indicate PCR products. (E) EMSA demonstrates the binding of rCCN2 to the proximal regulatory region of the *Spi1* regulatory region. The biotin‐labeled probe was made with a PCR technique using genomic DNA derived from the NIH3T3 cells as a template and specific primers amplifying the proximal region in the *Spi1* promoter. After rCCN2 was allowed to interact with the biotin‐labeled probe or with the biotin‐labeled probe and unlabeled probe, electrophoresis was carried out. BSA was used as a negative control. The shifted band of the rCCN2 complex was detected and was diminished by the co‐treatment with unlabeled probe. No complex was detected with BSA. (F) The shifted band included rCCN2. The membrane shown in panel (E) was re‐blotted using an anti‐CCN2 antibody. The shifted band shown in panel (E) showed immunoreactivity for CCN2 (arrow). BSA, bovine serum albumin; CCN2, cellular communication network factor 2; ChIP‐PCR, chromatin immunoprecipitation PCR; EMSA, electrophoretic mobility shift assay; EV, empty vector; PU.1, purine‐rich box 1; rCCN2, recombinant CCN2; and RT‐PCR, reverse transcription polymerase chain reaction.

To clarify the effect of the translocation of CCN2 into the nucleus on *Spi1* expression, we firstly investigated the gene expression of *Spi1* in NIH3T3 cells transfected with p*CCN2*‐HA. As shown in Figure 2A, the gene expression level of *Spi1* was significantly upregulated by the transfection with p*CCN2*‐HA, compared to that by the transfection with an EV. Next, to test the regulatory mechanism of *Spi1* expression due to transfection with p*CCN2*‐HA, we performed ChIP‐PCR analysis, using specific primers to detect distal and proximal regulatory regions that were previously reported to be highly conserved among species (Figure 2B).^35^ As a result, we observed a distinct band, which indicates the binding of CCN2 to the regulatory element in the proximal region of *Spi1* (Figure 2D; see the arrow), but no band indicating binding to the distal element (Figure 2C; see the arrow). These data indicate that CCN2 binds to the proximal regulatory region of *Spi1*. Therefore, to confirm this result, we performed EMSA using a biotin‐labeled probe derived from the proximal region of *Spi1*. After the reaction of rCCN2 and the biotin‐labeled probe at 4°C overnight, electrophoresis was performed. As shown in Figure 2E, the band shifted upwards in the sample with the rCCN2 addition (see the arrow at the top), but the shifted band was not observed in the sample with the BSA addition as a negative control. To confirm the specificity of this shifted band, we added the unlabeled probe as a competitor to the sample containing rCCN2 and biotin‐labeled probe, and we showed that the shifted band was diminished (Figure 2E; see the arrow at the top). Next, to confirm that rCCN2 is involved in this shifted band, we re‐blotted the membrane shown in Figure 2E using an anti‐CCN2 antibody. As a result, the shifted band shown in Figure 2E displayed immunoreactivity for CCN2 (Figure 2F; see the arrow). Collectively, it was clearly indicated that CCN2 bound to the *Spi1* regulatory element in the proximal region.

### 3.3 CCN2 interacts with PU.1 in the nucleus

**FIGURE 3:**
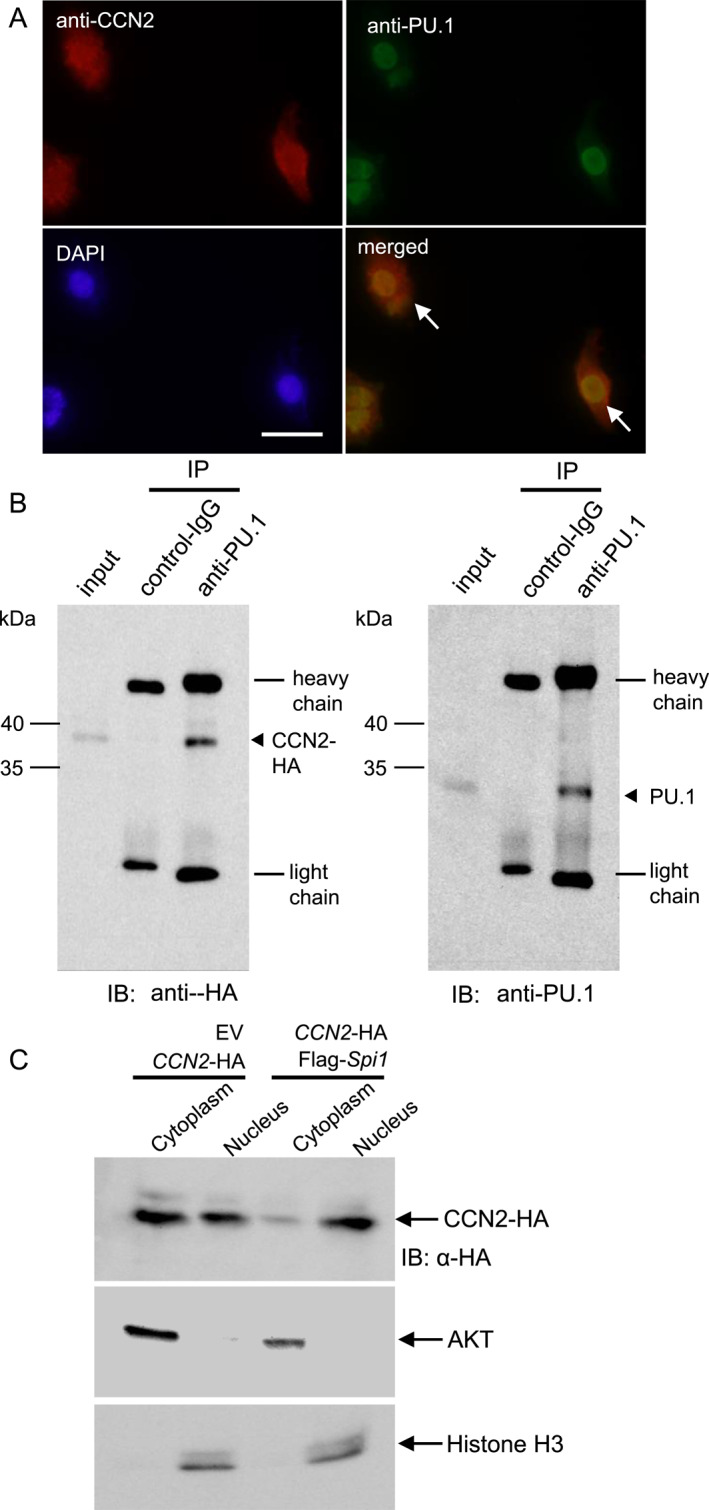
Interaction between CCN2 and PU.1 in the nucleus. (A) Indirect immunofluorescence analysis of CCN2 and PU.1. After simultaneous transfection with p*CCN2*‐HA and pFlag‐*Spi1*, NIH3T3 cells were cultured for 48 h. An indirect immunofluorescence analysis was then performed with anti‐CCN2 and anti‐PU.1 antibodies. The co‐localization of CCN2 and PU.1 was detected in the nucleus. The arrows in the merged image indicate the co‐localization of CCN2 and PU.1. The bar represents 30 μm. (B) Interaction of CCN2 with PU.1. Forty‐eight hours after transfection with both p*CCN2*‐HA and pFlag‐*Spi1*, a nuclear extract was prepared from cell lysate. The input samples of 10% volume were taken from the cell lysate, and the remaining samples were immunoprecipitated with an anti‐PU.1 antibody or non‐immune control IgG. Next, a Western blot analysis was performed by using anti‐HA (left‐hand panel) and anti‐PU.1 antibodies (right‐hand panel). The arrowheads in the left and right panel indicate CCN2 conjugated with HA tag and PU.1, respectively. (C) Western blot analysis of CCN2 using NIH3T3 cells with the forced expression of CCN2 and PU.1. After transfection, the cell lysate was prepared and separated into cytoplasmic and nuclear fractions. A Western blot analysis was then performed. AKT and Histone H3 are markers of the cytoplasm and nucleus, respectively. Positions of the molecular weight markers are shown at the left. CCN2, cellular communication network factor 2 and PU.1, purine‐rich box 1.

Because CCN2 controls *Spi1* expression, we next investigated whether or not CCN2 interacts with PU.1 in the nucleus. As shown in Figure 3A, we examined the localization of CCN2 and PU.1 in NIH3T3 cells overexpressing both CCN2 and PU.1 using immunofluorescence analysis. As a result, CCN2 was found to be co‐localized with PU.1 in the nucleus of the NIH3T3 cells (merged image; see the arrows). This finding indicates the possible interaction of CCN2 with PU.1 in the nucleus of NIH3T3 cells. Next, to examine this possibility, we performed IP‐Western blot analysis using the nuclear extract from the NIH3T3 cells with the forced expression of both CCN2 and PU.1. After confirming that PU.1 was immunoprecipitated in the nuclear protein using an anti‐PU.1 antibody, a Western blot analysis was carried out using an anti‐HA antibody to detect CCN2. As shown in Figure 3B (left‐hand panel), immunoreactivity for HA was detected in the proteins immunoprecipitated from the nuclear extract of the cells with an anti‐PU.1 antibody, but it was not detected in the proteins immunoprecipitated with non‐immune control IgG. In addition, what was immunoprecipitated with anti‐PU.1 antibody is shown in Figure 3B; right‐hand panel. These results indicate that CCN2 physically interacts with PU.1 in the nucleus. Furthermore, we next examined whether or not the nuclear localization of CCN2 was facilitated by PU.1 co‐expression. To test this hypothesis, after p*CCN2*‐HA‐ and pFlag‐*Spi1*‐transfected NIH3T3 cell lysates were separated into their nuclear and cytoplasmic fractions, Western blot was performed using an anti‐HA antibody. Compared to the cell lysate with the forced expression of CCN2 only, immunoreactivity for CCN2 was increased in the nuclear fraction with the overexpression of both CCN2 and PU.1 (Figure 3C). This result suggests that CCN2's nuclear translocation is facilitated in the presence of PU.1.

### 3.4 The forced expression of both CCN2 and PU.1 controls the components from the RAS and fibrotic markers

**FIGURE 4:**
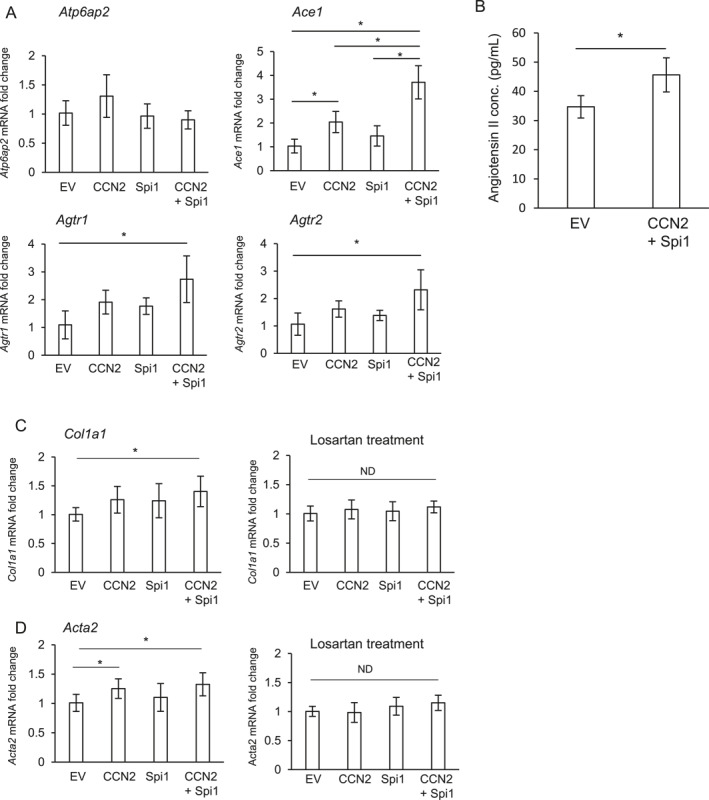
Gene expression of RAS components and fibrotic markers in NIH3T3 cells simultaneously transfected with p*CCN2*‐HA and pFlag‐*Spi1*. (A) The gene expression levels of RAS components in NIH3T3 cells transfected with p*CCN2*‐HA, pFlag‐*Spi1*, or both p*CCN2*‐HA and pFlag‐*Spi1*. After transfection, NIH3T3 cells were cultured for 48 h. The amount of *Gapdh* mRNA was used as a reference. The ΔΔCt method was used for the relative quantification of these gene expressions. The bars represent mean values and SDs from independent cultures (*n* = 6) and indicate the relative ratio with respect to the transfection with EV (EV = 1.0). The data were analyzed using Bonferroni's test following normality testing. The asterisk indicates a significant difference at *p* < 0.05. ND indicates no significant difference. (B) NIH3T3 cells were transfected with both p*CCN2*‐HA and pFlag‐*Spi1*, and after 48 h, the cell lysate was harvested. Quantification of ANG II was performed by using an ELISA system. The bars represent mean values and SDs from independent cultures (*n* = 4) and analyzed by Student's *t*‐test. The asterisk indicates a significant difference at *p* < 0.05. (C, D) Effect of Losartan on *Col1a1* (C) and *Acta2* (D) expressions in NIH3T3 cells transfected with both p*CCN2*‐HA and pFlag‐*Spi1*. After 24 h of transfection, NIH3T3 cells were treated with (right‐hand panel) or without (left‐hand panel) 50 μM losartan potassium salt and were further cultured for 24 h. The bars represent mean values and SDs from independent culture dishes (*n* = 6), which was computed by the ΔΔCt method. The ordinate of the graphs indicates the relative ratio with respect to the transfection with EV (EV = 1.0). The data were analyzed using Bonferroni's test following normality testing. The asterisk indicates a significant difference at *p* < 0.05. ND indicates no significant difference. ELISA, enzyme‐linked immunosorbent assay; EV, empty vector; RAS, renin‐angiotensin system; and SDs, standard deviations.

**FIGURE 5:**
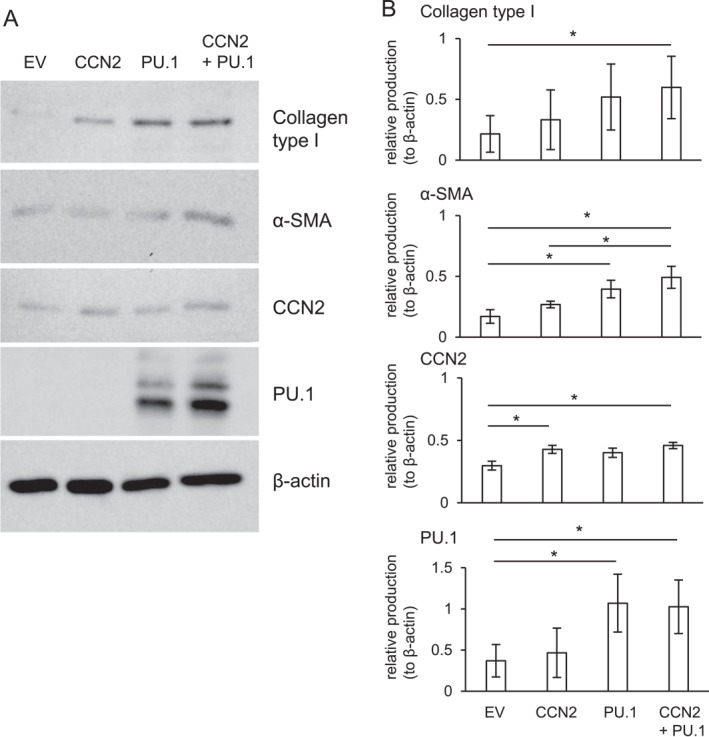
Western blot analysis of type I collagen, α‐SMA, CCN2, and PU.1 productions in NIH3T3 cells simultaneously transfected with p*CCN2*‐HA and pFlag‐*Spi1*. (A) NIH3T3 cells were transfected with p*CCN2*‐HA, pFlag‐*Spi1*, or both p*CCN2*‐HA and pFlag‐*Spi1* by electroporation, and, 3 days later, a Western blot analysis was performed using anti‐type I collagen, α‐SMA, CCN2, and PU.1 antibodies. As a loading control, the production of β‐actin was also analyzed. Positions of the molecular weight markers are shown at the left. (B) The amounts of these productions were determined densitometrically and these amounts were normalized to the amount of β‐actin. All graphs show the mean (±SD) of the relative ratio and the asterisks indicate significant differences as revealed by Bonferroni's test (*p* < 0.05). α‐SMA, α‐smooth muscle actin; CCN2, cellular communication network factor 2; and PU.1, purine‐rich box 1; SD, standard deviation.

Next, we investigated how the interaction of CCN2 with PU.1 play a role in fibrosis. It is well known that ANG II, which is locally produced from the renin‐angiotensin system (RAS), promotes fibrosis.^36^ Therefore, we examined the gene expression levels of RAS components. Although the expression of *Atp6ap2* gene, which codes the prorenin receptor, showed no change, the gene expression level of angiotensin‐converting enzyme 1 (*Ace1*), which is an enzyme that converts angiotensin I to ANG II, was significantly upregulated in the cells with the forced expression of both CCN2 and PU.1, compared to that with EV or CCN2 only (Figure 4A). In addition, ANG II receptors, *Agtr1* and *Agtr2*, were also increased by the overexpression of both CCN2 and PU.1 (Figure 4A). Next, we investigated whether or not the production of ANG II is increased by the forced expression of both CCN2 and PU.1 by using ANG II ELISA kit (Enzo Life Sciences). As shown in Figure 4B, the production of ANG II was significantly increased in the cell lysate of NIH3T3 cells transfected with both p*CCN2*‐HA and pFlag‐*Spi1*. These findings indicate that the forced expression of CCN2 and PU.1 controls ANG II production and its functions, thus suggesting that they promote fibrosis. Indeed, the expressions of *Col1a1* and *Acta2*, which are fibrotic markers, were increased with the combination of CCN2 and PU.1, compared with those with EV (Figure 4C left‐hand panel). Interestingly, the upregulation of these genes was disappeared by the treatment with losartan potassium salt, which is a blocker of ANG II receptor type I (Figure 4C right‐hand panel). These results suggest that ANG II produced by the co‐expression of CCN2 and PU.1 increases the fibrosis‐related gene expressions via the interaction with ANG II receptor type I. Next, we examined the effect of a combination of CCN2 and PU.1 on the productions of type I collagen, α‐SMA, CCN2, and PU.1. As shown in Figure 5A,B, the production of type I collagen and α‐SMA were increased by the forced expression of both CCN2 and PU.1, compared with that of EV. On the other hand, the production levels of CCN2 or PU.1 by the forced expression of both plasmids had no effect, compared with the production level by the forced expression of each plasmid (Figure 5A,B). These findings suggest that increased productions of both factors by the simultaneous forced expression of CCN2 and PU.1 control fibrosis‐related factors, such as type I collagen and α‐SMA, at the protein level as well.

## 4 DISCUSSION

In the present study, we demonstrated that CCN2 was translocated into the nucleus in fibroblasts and that it regulates the gene expression and protein production of type I collagen and α‐SMA, which are markers of fibrosis (Figures 4 and 5). These results indicate that CCN2 has intracrine effects. Although it is well known that transcription factor PU.1, which promotes and suppresses the differentiation of hematopoietic stem cells to B cells and to pre‐erythroblasts, respectively,^5^ it was re‐discovered that PU.1 is involved in the pathological conditions of fibrosis.^6^ Namely, the report demonstrated that PU.1 was a transcription factor regulating differentiation to myofibroblasts. However, it is still unknown how the production of PU.1 is regulated under fibrotic conditions. In the present study, we showed that CCN2, which is reported as a promoter of fibrosis, is translocated into the nucleus in fibroblasts and binds to the regulatory region that is conserved among various species, which is located approximately −14 kb upstream from the initiation codon of *Spi1* (Figure 2B,C). Additionally, our data also revealed that the gene expression level of *Spi1* is significantly increased by transfection with p*CCN2*‐HA (Figure 2A). These results indicate that CCN2 translocated into the nucleus is important for controlling *Spi1* gene expression, suggesting that CCN2 works as a transcription co‐factor.

In another report, it was also described that CCN2 translocates into the nuclei of human mesangial cells.^24^ According to this report, when radio‐labeled CCN2 is added from outside the cells, radioisotope activities are detected in the cytoplasm within 10 min and in the nucleus within 30 min.^24^ Moreover, another study showed that CCN2 was internalized into the cells via low density lipoprotein receptor‐related protein 1 (LRP1).^37^ Based on these reports, it is suspected that, after CCN2 is secreted into the extracellular environment, secreted CCN2 is re‐incorporated into the cytoplasm via LRP1 and then translocated into the nucleus. Based on this speculation, we hypothesized that soluble heparin, which binds to CCN2 strongly, disturbs the re‐incorporation of CCN2; as a result, the nuclear localization of CCN2 is decreased. However, the treatment with soluble heparin from the outside of cells was not able to disrupt the localization of CCN2 into the nucleus (Figure S1). In addition, the gene expression level of *Spi1* was significantly increased by transfection with p*CCN2*‐HA (Figure 2A), and the protein production level of PU.1 was also shown to be increased (Figure S2A), although it was the results obtained by a longer exposure of Western blotting. On the other hand, it showed no change in the treatment with rCCN2 (Figure S2B). These results indicate that the secretion of CCN2 to outside the cells may be not involved in the translocation of CCN2 into the nucleus, but rather the intracellular CCN2 may be involved in it. We showed that, in the cells with the simultaneous forced expression of PU.1 and CCN2, CCN2's nuclear translocation was enhanced, compared with that in the cells with forced expression of CCN2 only (Figure 3C). We also detected that CCN2 bound to PU.1 physically (Figure 3B). These results indicate that PU.1 may escort CCN2 into the nucleus. However, further investigation is needed to clarify the mechanism of CCN2's translocation into the nucleus.

As it has been reported that ANG II promotes fibrosis through the control of the expression of PU.1,^38^ we considered that this interaction between PU.1 and CCN2 may also affect the gene expressions of the components of RAS in a positive feedback manner. Thus, we investigated the gene expression levels of RAS components, such as *Atp6qp2*, *Ace1*, *Agtr1*, and *Agtr2*. Interestingly, we showed that *Ace1* expression was upregulated more in the NIH3T3 cells with the forced expression of both PU.1 and CCN2 than in those with the forced expression of *CCN2* or PU.1 only (Figure 4A). Additionally, the gene expressions of not only *Agtr1* and *Agtr2*, which are ANG II receptors, but also of *Col1a1* and *Acta2* were also upregulated by the transfection of both *CCN2* and *Spi1* genes, compared to those affected by the transfection with EV (Figure 4A). Furthermore, CCN2 in combination with PU.1 significantly increased ANG II production (Figure 4B). These findings suggest that this complex is involved in the progression of fibrosis via promoting ANG II production.

To investigate the origin of myofibroblasts, fibroblasts derived from mice with myocardial infarction injury via the surgical ligation of the left coronary artery have been recently characterized by using a method from a lineage‐tracing experiment.^39^ This report clarified that the fibroblasts termed activated fibroblasts, which had high proliferation activity, invaded the necrotic area within 2–4 days of infarction injury, secreted new ECMs, and remodeled the ECMs.^39^ Moreover, within 3–7 days, these cells were shown to differentiate from activated fibroblasts into myofibroblasts that secreted abundant ECM components such as α‐SMA and type I collagen to structurally support the necrotic area.^39^ Finally, within 7–10 days, the myofibroblasts were observed to lose proliferative activity, and the scar fully matured.^39^ These findings suggest that fibroblasts have the ability to transdifferentiate into various forms under cellular stress, such as infarction. Furthermore, single‐cell RNA‐sequencing analysis using heart cells isolated from mice with experimentally induced cardiac disease revealed six major clusters of cardiomyocytes, endothelial cells, fibroblasts, macrophages, T cells, and granulocytes.^40^ Moreover, it was shown that the fibroblast clusters from this cardiac disease model comprise nine distinct subtypes.^40^ Among them, group 1 comprised the cell population of activated fibroblasts, and group 6 comprised that of myofibroblasts.^40^ These findings indicate that fibrotic lesions are heterogeneous tissues consisted with many types of fibroblasts producing multiple signaling factors, thus suggesting that the progression of fibrosis is controlled by such factors. In particular, evidence showing the role of CCN2 in fibrosis using in vivo and in vitro models is increasing.^41^, ^42^, ^43^ On the other hand, some of these reports have shown that CCN2 only has a weak effect on progression of fibrosis.^44^, ^45^, ^46^ Namely, although CCN2 is induced by profibrotic factors, such as transforming growth factor (TGF)‐β^47^, ^48^ and is secreted to outside of the cells, secreted CCN2 may work to create an extracellular environment favorable for fibrotic conditions without playing a central role in fibrosis. Rather, CCN2 may a more important role in the progression of fibrosis, after interacting with PU.1 and being translocated into the nucleus. Consequently, nuclear‐translocated CCN2 could upregulate the expressions of several fibrotic‐related genes in cooperation with PU.1.

## 5 CONCLUSION

We clarified that CCN2 is translocated into the nuclei of fibroblasts and the nuclear‐translocated CCN2 binds to the regulatory region of *Spi1* expression. Our data also showed that CCN2 interacts with PU.1 to facilitate the nuclear translocation. Furthermore, this complex cooperatively increased the fibrosis‐related molecules, such as RAS components, type I collagen, and α‐SMA. Taken together, these findings suggest that nuclear‐translocated CCN2 forms the complex of PU.1 and positively regulates fibrotic‐related factors. However, more investigation is needed to clarify the detailed mechanism of the action of CCN2 as a transcription co‐factor.

## AUTHOR CONTRIBUTIONS

**Xuan Thi Nguyen**: Writing—original draft; investigation; formal analysis; data curation. **Satoshi Kubota**: Writing—review and editing; supervision; funding acquisition; validation; project administration. **Masaharu Takigawa**: Writing—review and editing; funding acquisition; validation. **Takashi Nishida**: Writing—original draft; supervision; investigation; data curation; formal analysis; validation; project administration; methodology; visualization; funding acquisition; conceptualization.

## CONFLICT OF INTEREST STATEMENT

The authors declare no conflicts of interest.

## ETHICS STATEMENT

This study did not require any ethics approval.

## Supporting information

Supporting Information S1

Supporting Information S2

Figure S1

Figure S2

Table S1

Table S2

## Data Availability

Data generated and/or analyzed during this study are available from the corresponding author on request.

## References

[1] Wynn, T. A. , and T. R. Ramalingam . 2012. “Mechanisms of Fibrosis: Therapeutic Translation for Fibrotic Disease.” Nature Medicine 18(7): 1028–1040. 10.1038/nm.2807. PMC3405917 22772564

[2] Weiskirchen, R. , S. Weiskirchen , and F. Tacke . 2019. “Organ and Tissue Fibrosis: Molecular Signals, Cellular Mechanisms and Translational Implications: (Review).” Molecular Aspects of Medicine 65: 2–15. 10.1016/j.mam.2018.06.003. 29958900

[3] Wynn, T. A. 2004. “Fibrotic Disease and the T_H_1/T_H_2 Paradigm.” Nature Reviews Immunology 4(8): 583–594. 10.1038/nri1412. PMC2702150 15286725

[4] Hu, B. , and S. H. Phan . 2013. “Myofibroblasts.” Current Opinion in Rheumatology 25(1): 71–77. 10.1097/BOR.0b013e32835b1352. 23114586 PMC4007118

[5] Karim, F. D. , L. D. Urness , C. S. Thummel , M. J. Klemsz , S. R. McKercher , A. C. Van Beveren , R. A. Maki , C. V. Gunther , and J. A. Nye . 1990. “The ETS‐Domain: A New DNA‐Binding Motif that Recognizes a Purine‐Rich Core DNA Sequence.” Genes & Development 4(9): 1451–1453. 10.1101/gad.4.9.1451. 2253872

[6] Wohlfahrt, T. , S. Rauber , S. Uebe , M. Luber , A. Soare , A. Ekici , S. Weber , et al. 2019. “PU.1 Controls Fibroblast Polarization and Tissue Fibrosis.” Nature 566(7744): 344–349. 10.1038/s41586-019-0896-x. 30700907 PMC6526281

[7] Sun, C. , H. Zhang , and X. Liu . 2021. “Emerging Role of CCN Family Proteins in Fibrosis (Review).” Journal of Cellular Physiology 236(6): 4195–4206. 10.1002/jcp.30171. 33222181

[8] Fadl, A. , and A. Leask . 2025. “CCN2: A Potential Contributor to Gingival Overgrowth (Review).” Journal of Oral Biosciences 67(1): 100587. 10.1016/j.job.2024.100587. 39521130

[9] Perbal, B. , and M. Takigawa . 2005. “The CCN Family of Proteins: An Overview” CCN Proteins: A New Family of Cell Growth and Differentiation Regulators, 1–18. London: Imperial College Press.

[10] Takigawa, M. 2013. “CCN2: A Master Regulator of the Genesis of Bone and Cartilage.” Journal of Cell Communication and Signaling 7(3): 191–201. 10.1007/s12079-013-0204-8. 23794334 PMC3709051

[11] Holbourn, K. P. , K. R. Acharya , and B. Perbal . 2008. “The CCN Family of Proteins: Structure‐Function Relationships.” Trends in Biochemical Sciences 33(10): 461–473. 10.1016/j.tibs.2008.07.006. 18789696 PMC2683937

[12] Chen, N. , C.‐C. Chen , and L. F. Lau . 2000. “Adhesion of Human Skin Fibroblasts to Cyr61 Is Mediated Through Integrin α6β1 and Cell Surface Heparan Sulfate Proteoglycans.” Journal of Biological Chemistry 275(32): 24953–24961. 10.1074/jbc.M003040200. 10821835

[13] Todorovic, V. , C.‐C. Chen , N. Hay , and L. F. Lau . 2005. “The Matrix Protein CCN1 (CYR61) Induces Apoptosis in Fibroblasts.” Journal of Cell Biology 171(3): 559–568. 10.1083/jcb.200504015. 16275757 PMC1626352

[14] Jun, J.‐l. , and L. F. Lau . 2010. “The Matricellular Protein CCN1 Induces Fibroblast Senescence and Restricts Fibrosis in Cutaneous Wound Healing.” Nature Cell Biology 12(7): 676–685. 10.1038/ncb2070. 20526329 PMC2919364

[15] Jun, J.‐l. , and L. F. Lau . 2010. “Cellular Senescence Controls Fibrosis in Wound Healing.” Aging (Albany NY) 2(9): 627–631. 10.18632/aging.100201. 20930261 PMC2984611

[16] Abd El Kader, T. , S. Kubota , D. Janune , T. Nishida , T. Hattori , E. Aoyama , B. Perbal , T. Kuboki , and M. Takigawa . 2013. “Anti‐Fibrotic Effect of CCN3 Accompanied by Altered Gene Expression Profile of the CCN Family.” Journal of Cell Communication and Signaling 7(1): 11–18. 10.1007/s12079-012-0180-4. 23065484 PMC3590363

[17] Riser, B. L. , F. Najmabadi , B. Perbal , D. R. Peterson , J. A. Rambow , M. L. Riser , E. Sukowski , H. Yeger , and S. C. Riser . 2009. “CCN3 (NOV) Is a Negative Regulator of CCN2 (CTGF) and a Novel Endogenous Inhibitor of the Fibrotic Pathway in an In Vitro Model of Renal Disease.” American Journal of Pathology 174(5): 1725–1734. 10.2353/ajpath.2009.080241. 19359517 PMC2671261

[18] Yoon, P. O. , M.‐A. Lee , H. Cha , M. H. Jeong , J. Kim , S. P. Jang , B. Y. Choi , et al. 2010. “The Opposing Effects of CCN2 and CCN5 on the Development of Cardiac Hypertrophy and Fibrosis.” Journal of Molecular and Cellular Cardiology 49(2): 294–303. 10.1016/j.yjmcc.2010.04.010. 20430035

[19] Batmunkh, R. , Y. Nishioka , Y. Aono , M. Azuma , K. Kinoshita , J. Kishi , H. Makino , M. Kishi , A. Takezaki , and S. Sone . 2011. “CCN6 as a Profibrotic Mediator that Stimulates the Proliferation of Lung Fibroblasts via the Integrin β1/Focal Adhesion Kinase Pathway.” Journal of Medical Investigation 58(3–4): 188–196. 10.2152/jmi.58.188. 21921419

[20] Song, Y. , C. Li , Y. Luo , J. Guo , Y. Kang , F. Yin , L. Ye , D. Sun , J. Yu , and X. Zhang . 2023. “CCN6 Improves Hepatic Steatosis, Inflammation, and Fibrosis in Non‐Alcoholic Steatohepatitis.” Liver International 43(2): 357–369. 10.1111/liv.15430. 36156376

[21] Jian, Y.‐C. , J.‐J. Wang , S. Dong , J.‐W. Hu , L.‐J. Hu , G.‐M. Yang , Y.‐X. Zheng , and W.‐J. Xiong . 2014. “Wnt‐Induced Secreted Protein 1/CCN4 in Liver Fibrosis Both In Vitro and In Vivo.” Clinical Laboratory 60(1): 29–35. 10.7754/clin.lab.2013.121035. 24600972

[22] Leask, A. 2017. “CCN2 in Skin Fibrosis.” Methods in Molecular Biology 1489: 417–421. 10.1007/978-1-4939-6430-7_34. 27734393

[23] Richeldi, L. , E. R. Fernández Pérez , U. Costabel , C. Albera , D. J. Lederer , K. R. Flaherty , N. Ettinger , et al. 2020. “Pamrevlumab, an Anti‐Connective Tissue Growth Factor Therapy, for Idiopathic Pulmonary Fibrosis (PRAISE): A Phase 2, Randomized, Double‐Blind, Placebo‐Controlled Trial.” The Lancet Respiratory Medicine 8(1): 25–33. 10.1016/S2213-2600(19)30262-0. 31575509

[24] Wahab, N. A. , H. Brinkman , and R. M. Mason . 2001. “Uptake and Intracellular Transport of the Connective Tissue Growth Factor: A Potential Mode of Action.” Biochemical Journal 359(Pt1): 89–97. 10.1042/0264-6021:3590089. 11563972 PMC1222124

[25] Hutchenreuther, J. , K. M. Vincent , D. E. Carter , L.‐M. Postovit , and A. Leask . 2015. “CCN2 Expression by Tumor Stroma Is Required for Melanoma Metastasis.” Journal of Investigative Dermatology 135(11): 2805–2813. 10.1038/jid.2015.279. 26168233

[26] Re, R. N. , and J. L. Cook . 2008. “The Basis of an Intracrine Pharmacology.” The Journal of Clinical Pharmacology 48(3): 344–350. 10.1177/0091270007312155. 18223145

[27] Raghu, G. , L. Richeldi , E. R. Fernández Pérez , M. C. De Salvo , R. S. Silva , J. W. Song , T. Ogura , et al. 2024. “Pamrevlumab for Idiopathic Pulmonary Fibrosis: The ZEPHYRUS‐1 Randomized Clinical Trial.” Journal of the American Medical Association 332(5): 380–383. 10.1001/jama.2024.8693. 38762797 PMC11304118

[28] Nishida, T. , S. Kubota , and M. Takigawa . 2017. “Production of Recombinant CCN2 Protein by Mammalian Cells.” Methods in Molecular Biology 1489: 95–105. 10.1007/978-1-4939-6430-7_10. 27734369

[29] Nishida, T. , S. Akashi , M. Takigawa , and S. Kubota . 2021. “Effect of Angiotensin II on Chondrocyte Degeneration and Protection via Differential Usage of Angiotensin II Receptors.” International Journal of Molecular Sciences 22(17): 9204. 10.3390/ijms22179204. 34502113 PMC8430521

[30] Hochi, H. , S. Kubota , M. Takigawa , and T. Nishida . 2023. “Dual Roles of Cellular Communication Network Factor 6 (CCN6) in the Invasion and Metastasis of Oral Cancer Cells to Bone via Binding to BMP2 and RANKL.” Carcinogenesis 44(8–9): 695–707. 10.1093/carcin/bgad057. 37590989 PMC10692700

[31] Nishida, T. , Y. Nagao , S. Hashitani , N. Yamanaka , M. Takigawa , and S. Kubota . 2020. “Suppression of Adipocyte Differentiation by low‐intensity Pulsed Ultrasound via Inhibition of Insulin Signaling and Promotion of CCN Family Protein 2.” Journal of Cellular Biochemistry 121(12): 4724–4740. 10.1002/jcb.29680. 32065439

[32] Livak, K. J. , and T. D. Schmittgen . 2001. “Analysis of Relative Gene Expression Data Using Real‐Time Quantitative PCR and the 2^‐ΔΔCt^ Method.” Methods 25(4): 402–408. 10.1006/meth.2001.1262. 11846609

[33] Nishida, T. , S. Kubota , E. Aoyama , D. Janune , A. Maeda , and M. Takigawa . 2011. “Effect of CCN2 on FGF2‐Induced Proliferation and MMP9 and MMP13 Productions by Chondrocytes.” Endocrinology 152(11): 4232–4241. 10.1210/en.2011-0234. 21914781

[34] Eguchi, T. , S. Kubota , K. Kawata , Y. Mukudai , J. Uehara , T. Ohgawara , S. Ibaragi , A. Sasaki , T. Kuboki , and M. Takigawa . 2008. “Novel Transcription‐Factor‐Like Function of Human Matrix Metalloproteinase 3 Regulating the CTGF/CCN2 Gene.” Molecular and Cellular Biology 28(7): 2391–2413. 10.1128/MCB.01288-07. 18172013 PMC2268440

[35] Okuno, Y. , G. Huang , F. Rosenbauer , E. K. Evans , H. S. Radomska , H. Iwasaki , K. Akashi , et al. 2005. “Potential Autoregulation of Transcription Factor PU.1 by an Upstream Regulatory Element.” Molecular and Cellular Biology 25(7): 2832–2845. 10.1128/MCB.25.7.2832-2845.2005. 15767686 PMC1061634

[36] Schultz, J. E. J. , S. A. Witt , B. J. Glascock , M. L. Nieman , P. J. Reiser , S. L. Nix , T. R. Kimball , and T. Doetschman . 2002. “TGF‐β1 Mediates the Hypertrophic Cardiomyocyte Growth Induced by Angiotensin II.” Journal of Clinical Investigation 109(6): 787–796. 10.1172/JCI14190. 11901187 PMC150912

[37] Segarini, P. R. , J. E. Nesbitt , D. Li , L. G. Hays , J. R. Yates III , and D. F. Carmichael . 2001. “The Low Density Lipoprotein Receptor‐Related Protein/α2‐Macroglobuline Receptor Is a Receptor for Connective Tissue Growth Factor.” Journal of Biological Chemistry 276(44): 40659–40667. 10.1074/jbc.M105180200. 11518710

[38] Hu, J. , J.‐J. Zhang , L. Li , S.‐L. Wang , H.‐T. Yang , X.‐W. Fan , L.‐M. Zhang , et al. 2021. “PU.1 Inhibition Attenuates Atrial Fibrosis and Atrial Fibrillation Vulnerability Induced by Angiotensin‐II by Reducing TGF‐β1/Smads Pathway Activation.” Journal of Cellular and Molecular Medicine 25(14): 6746–6759. 10.1111/jcmm.16678. 34132026 PMC8278085

[39] Fu, X. , H. Khalil , O. Kanisicak , J. G. Boyer , R. J. Vagnozzi , B. D. Maliken , M. A. Sargent , et al. 2018. “Specialized Fibroblast Differentiated States Underlie Scar Formation in the Infarcted Mouse Heart.” Journal of Clinical Investigation 128(5): 2127–2143. 10.1172/JCI98215. 29664017 PMC5957472

[40] Ren, Z. , P. Yu , D. Li , Z. Li , Y. Liao , Y. Wang , B. Zhou , and Li Wang . 2020. “Single‐Cell Reconstruction of Progression Trajectory Reveals Intervention Principles in Pathological Cardiac Hypertrophy.” Circulation 141(21): 1704–1719. 10.1161/CIRCULATIONAHA.119.043053. 32098504

[41] Lasky, J. A. , L. A. Ortiz , B. Tonthat , G. W. Hoyle , M. Corti , G. Athas , G. Lungarella , A. Brody , and M. Friedman . 1998. “Connective Tissue Growth Factor mRNA Expression Is Upregulated in Bleomycin‐Induced Lung Fibrosis.” American Journal of Physiology ‐ Lung Cellular and Molecular Physiology 275(2): L365–L371. 10.1152/ajplung.1998.275.2.L365. 9700098

[42] Rachfal, A. W. , and D. R. Brigstock . 2003. “Connective Tissue Growth Factor (CTGF/CCN2) in Hepatic Fibrosis.” Hepatology Research 26(1): 1–9. 10.1016/s1386-6346(03)00115-3. 12787797

[43] Liu, S. , X. Shi‐Wen , D. J. Abraham , and A. Leask . 2011. “CCN2 Is Required for Bleomycin‐Induced Skin Fibrosis in Mice.” Arthritis & Rheumatism 63(1): 239–246. 10.1002/art.30074. 20936632

[44] Kennedy, L. , S. Liu , X. Shi‐Wen , Y. Chen , M. Eastwood , M. Sabetkar , D. E. Carter , C. M. Black , D. J. Abraham , and A. Leask . 2007. “CCN2 Is Necessary for the Function of Mouse Embryonic Fibroblasts.” Experimental Cell Research 313(5): 952–964. 10.1016/j.yexcr.2006.12.006. 17239853

[45] Hayata, N. , Y. Fujio , Y. Yamamoto , T. Iwakura , M. Obana , M. Takai , T. Mohri , S. Nonen , M. Maeda , and J. Azuma . 2008. “Connective Tissue Growth Factor Induces Cardiac Hypertrophy Through Akt Signaling.” Biochemical and Biophysical Research Communications 370(2): 274–278. 10.1016/j.bbrc.2008.03.100. 18375200

[46] Leask, A. 2010. “Potential Therapeutic Targets for Cardiac Fibrosis: TGF‐β, Angiotensin, Endothelin, CCN2, and PDGF, Partners in Fibroblast Activation.” Circulation Research 106(11): 1675–1680. 10.1161/CIRCRESAHA.110.217737. 20538689

[47] Mori, T. , S. Kawara , M. Shinozaki , N. Hayashi , T. Kakinuma , A. Igarashi , M. Takigawa , T. Nakanishi , and K. Takehara . 1999. “Role and Interaction of Connective Tissue Growth Factor with Transforming Growth Factor‐β in Persistent Fibrosis: A Mouse Fibrosis Model.” Journal of Cellular Physiology 181(1): 153–159. 10.1002/(SICI)1097-4652(199910)181:1<153::AID-JCP16>3.0.CO;2-K. 10457363

[48] Leask, A. 2008. “Targeting the TGF‐β, Endothelin‐1 and CCN2 Axis to Combat Fibrosis in Scleroderma (Review).” Cellular Signalling 20(8): 1409–1414. 10.1016/j.cellsig.2008.01.006. 18296024

