# Supplementary material for: Interaction between nuclear‐translocated cellular communication network factor 2 and purine‐rich box 1 regulates the expression of fibrosis‐related genes
Source: J Cell Commun Signal. 2025 Sep 25;19(4):e70051. doi: 10.1002/ccs3.70051 (PMC12463490; doi:10.1002/ccs3.70051)
Supplement: Supplementary file 1 — Supporting Information S1 [file CCS3-19-e70051-s004.docx]

**Supplementary Fig. 1 caption**

**Treatment with heparin from outside of NIH3T3 cells does not affect CCN2’s translocation into the nucleus.** NIH3T3 cells were transfected with pCCN2-HA, and soluble heparin (100 μg/mL) was added to the culture 2 h later. The next day, the media were changed, and soluble heparin was added again. A total of 48 h after the transfection with p*CCN2*-HA, the cell lysate was collected, and cytoplasmic and nuclear fractions were prepared by distributing them from the cell lysate. A Western blot analysis was then performed. AKT and Histone H3 indicate the markers for the cytoplasm and nucleus, respectively. Positions of the molecular weight markers are shown at the left.
