# Supplementary material for: Interaction between nuclear‐translocated cellular communication network factor 2 and purine‐rich box 1 regulates the expression of fibrosis‐related genes
Source: J Cell Commun Signal. 2025 Sep 25;19(4):e70051. doi: 10.1002/ccs3.70051 (PMC12463490; doi:10.1002/ccs3.70051)
Supplement: Supplementary file 2 — Supporting Information S2 [file CCS3-19-e70051-s005.docx]

**Supplementary Fig. 2 caption**

**Production of PU.1 by the transfection with p*CCN2*-HA, and gene expression of *Spi1* in NIH3T3 cells added to exogenous recombinant CCN2 (rCCN2).** (A) NIH3T3 cells were transfected with p*CCN2*-HA or pFlag-*Spi1* by electroporation, and 3 days later, a Western blot analysis was performed using anti-PU.1 and β-actin antibodies. As a positive control, PU.1 production that obtained by the transfection with pFlag-*Spi1*, was also shown. PU.1 production by the forced expression of CCN2 was detected by a longer exposure. Positions of the molecular weight markers are shown at the left. (B) NIH3T3 cells were treated with rCCN2 (100 ng/mL) for 24 h, and the total RNA was then isolated. Quantitative RT-PCR analysis was performed using specific primers for *Spi1* and *Gapdh*. The amount of *Gapdh* mRNA was used as a reference, and the ordinate indicates the fold change with respect to the PBS-treated sample. The bars represent mean and SDs from independent culture dishes (n = 3), which was evaluated using the 2^-ΔΔCt^ method. A Student’s *t*-test was used for statistical analysis. None of the gene expression levels demonstrated significant differences.
