# Supplementary material for: Interaction between nuclear‐translocated cellular communication network factor 2 and purine‐rich box 1 regulates the expression of fibrosis‐related genes
Source: J Cell Commun Signal. 2025 Sep 25;19(4):e70051. doi: 10.1002/ccs3.70051 (PMC12463490; doi:10.1002/ccs3.70051)
Supplement: Supplementary file 3 — Figure S1 [file CCS3-19-e70051-s001.pptx]

## Slide 1
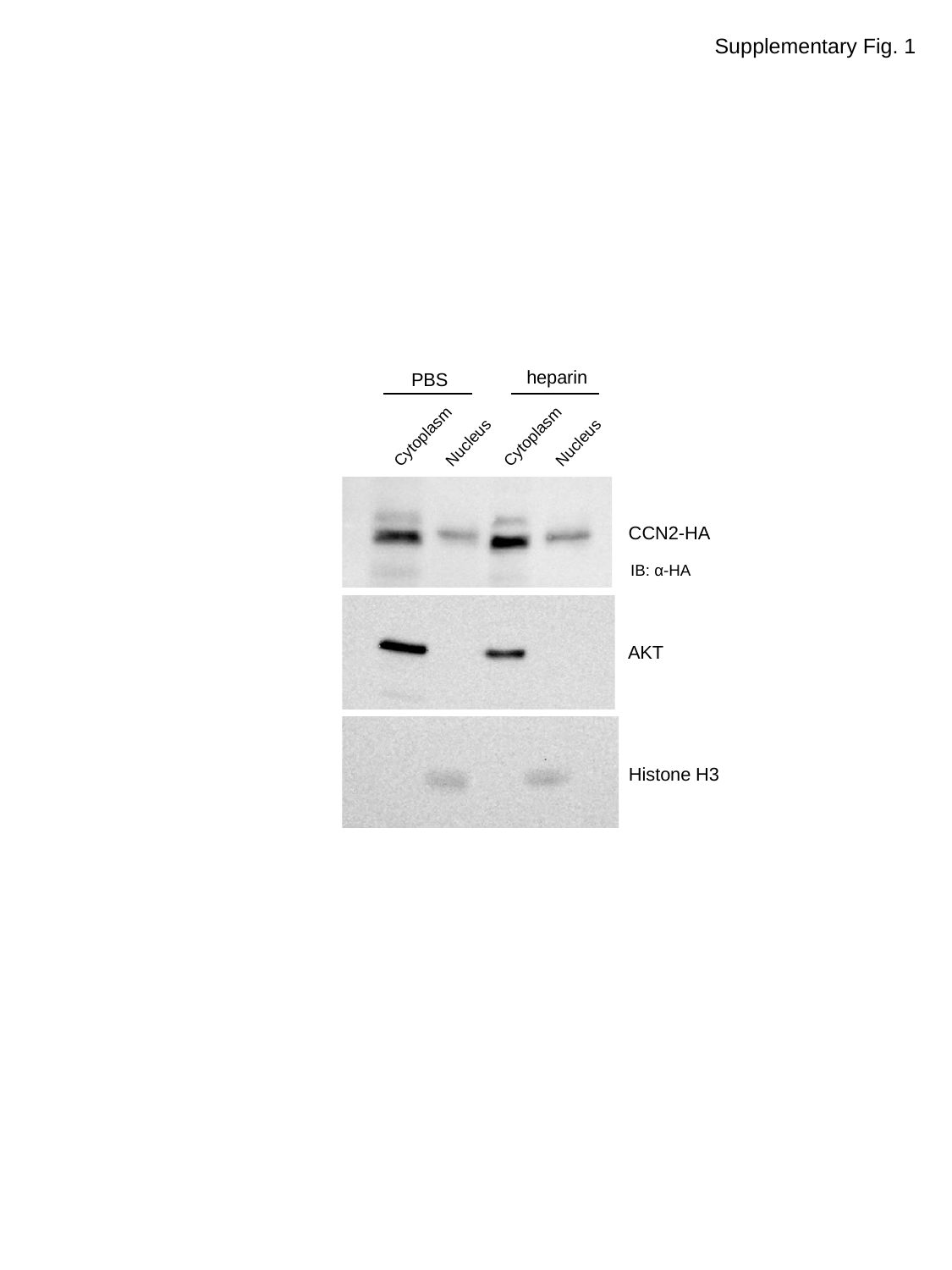

Supplementary Fig. 1
heparin
PBS
Cytoplasm
Cytoplasm
Nucleus
Nucleus
CCN2-HA
IB: α-HA
AKT
Histone H3
