# Supplementary figures and images for: Interaction between nuclear‐translocated cellular communication network factor 2 and purine‐rich box 1 regulates the expression of fibrosis‐related genes
Source: J Cell Commun Signal. 2025 Sep 25;19(4):e70051. doi: 10.1002/ccs3.70051 (PMC12463490; doi:10.1002/ccs3.70051)

## Slide 1
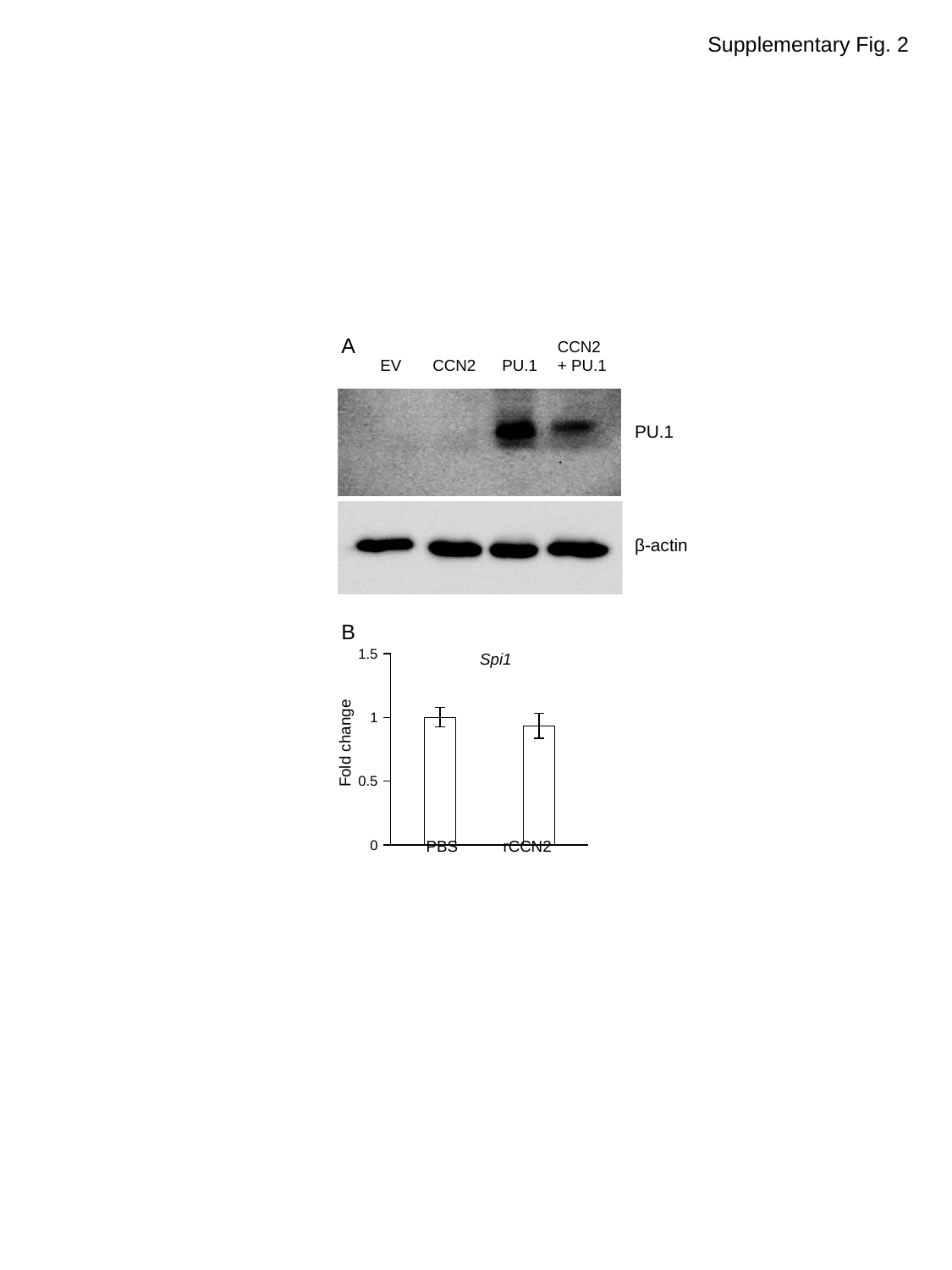

Supplementary Fig. 2
A
CCN2 + PU.1
EV
CCN2
PU.1
PU.1
β-actin
B
### Chart
| Category | |
|---|---| Spi1
Fold change
PBS
rCCN2

Supplement: Supplementary file 4 — Figure S2 [file CCS3-19-e70051-s003.pptx]
