# Supplementary material for: Interaction between nuclear‐translocated cellular communication network factor 2 and purine‐rich box 1 regulates the expression of fibrosis‐related genes
Source: J Cell Commun Signal. 2025 Sep 25;19(4):e70051. doi: 10.1002/ccs3.70051 (PMC12463490; doi:10.1002/ccs3.70051)
Supplement: Supplementary file 5 — Table S1 [file CCS3-19-e70051-s006.docx]

| Primary Antibody | Manufacturer | Identifier | Dilution ratio  WB IF IP |
| --- | --- | --- | --- |
| CCN2 | Proteintech | 25474-1-AP | 1:1000 |
| CCN2 | abcam | ab6992 | 1:100  (ChIP assay) |
| CCN2*  (anti-serum) |  |  | 1:100 |
| HA | Merck Millipore | 05-904 | 1:1000 1:300 |
| Flag | Cell Signaling technology | 14793S | 1:300 |
| Flag | Rockland Immunochemicals Inc. | 200-301-3835 | 1:500 |
| PU.1 | Santa-Cruz | sc-390405 | 1:100 1:100 1:20  (IP-Western) |
| AKT | Cell Signaling technology | 9272S | 1:1000 |
| Histone H3 | Epitomics Inc. | 1326-1 | 1:1000 |
| β-actin | Fujifilm Wako Pure Chemical | 010-27841 | 1:1000 |
| GST | Cytiva | 27457701V | 1:1000 |
| Collagen type I | Proteintech | 67288-1-Ig | 1:1000 |
| α-SMA | Epitomics Inc. | 1184-1 | 1:1000 |
| Rabbit non-immune control IgG | Agilent Dako | X0903 | 1:100  (ChIP assay) |
| Murine non-Immune control IgG | MillipreSigma | M9269 | 1:20  (IP-Western) |

Supplementary Table1. List of primary antibodies used in Western blot (WB) analysis, immunofluorescence (IF) analysis, and immunoprecipitation (IP)

*Anti-CCN2 serum: We prepared anti-CCN2 serum.
