# Supplementary material for: Interaction between nuclear‐translocated cellular communication network factor 2 and purine‐rich box 1 regulates the expression of fibrosis‐related genes
Source: J Cell Commun Signal. 2025 Sep 25;19(4):e70051. doi: 10.1002/ccs3.70051 (PMC12463490; doi:10.1002/ccs3.70051)
Supplement: Supplementary file 6 — Table S2 [file CCS3-19-e70051-s002.docx]

| Target Gene | Plasmid Name | Species | Primer Sequences |
| --- | --- | --- | --- |
| *Spi1* | pFlag-*Spi1* | mouse | (F) 5’-ggccgaattccTACCAACGTCCAATGCAT-3’  (R) 5’-atacggatccTCAGTGGGGCGGGAGGCGC-3’ |
| *GST* | pFlag-GST-NLS  pFlag-GST | schistosoma japonicum | (F) 5’-cccaagcttATGTCCCCTATACTAGGTTAT-3’  (R) 5’-ggcgaattcgCTTTTGGAGGATGGTCGCC-3’ |
| *NLS-like* | pFlag-GST-NLS | mouse | (F) 5’-atccgaattcAACATTAAGAAGGGCAAAAAGTGCATCCG-3’  (R) 5’-atacggatccGGATGCACTTTTTTGCCCTTCTTAATGTT-3’ |

Supplementary Table 2.

1. Sequences of forward (F) and reverse (R) primers used to create expression plasmids

(restriction site: lower cases).

| Gene | Accession No. | Species | Nucleotide Sequence |
| --- | --- | --- | --- |
| *Col1a1* | NM_007742.4 | mouse | (F) 5’-TCTCCACTCTTCTAGTTCCT-3’  (R) 5’-TTGGGTCATTTCCACATGC-3’ |
| *Acta2*  (α-SMA) | NM_007392.3 | mouse | (F) 5’-ACTGAGCGTGGCTATTCCTTCG-3’  (R) 5’-GCCGTGGCCATCTCATTTTCA-3’ |
| *Spi1*  (PU.1) | NM_001378899.1 | mouse | (F) 5’-ATGGAGAAAGCCATAGCGATCA-3’ (R) 5’-GACATGGTGTGCGGAGAAATC-3’ |
| *Atp6ap2* | NM_027439.4 | mouse | (F) 5’-CACATTGCGGCAGCTCCGTAA-3’  (R) 5’-CTCACAAGGGATGTGTCGAAT-3’ |
| *Ace1* | NM_207624.6 | mouse | (F) 5’-AACAAACATGATGGCCACATCCCG-3’  (R) 5’-CGTGTAGCCATTGAGCTTGGCAAT-3’ |
| *Agtr1* | NM_177322.3 | mouse | (F) 5’-CCATTGTCCACCCGATGAAG-3’  (R) 5’-TGCAGGTGACTTTGGCCAC-3’ |
| *Agtr2* | NM_007429.5 | mouse | (F) 5’-CAGCAGCCGTCCTTTTGATAA-3’  (R) 5’-TTATCTGATGGTTTGTGTGAGCAA -3’ |
| *Gapdh* | XM_011241214.1 | mouse | (F) 5’-GCCAAAAGGGTCATCATCTC-3’  (R) 5’-GTCTTCTGGGTGGCAGTGAT-3’ |

(B) Sequences of forward (F) and reverse (R) primers used for quantitative PCR.

|  | Forward (F) | Reverse (R) | length (bp) |
| --- | --- | --- | --- |
| *Spi1* distal | 5’-TGCCTGAGCTTCAGCAGAGATCTG-3’ | 5’-ACTCACCTCTCGTTCCTGGTCC-3’ | 305 |
| *Spi1* proximal | 5’-GCACACATGCTTCCTGTGGTGACT-3’ | 5’-CCATGTGCCCTAGCTGCTACCCCTA-3 | 362 |

(C) Sequences of forward (F) and reverse (R) primers used for ChIP-PCR.

|  | Forward (F) | Reverse (R) | length (bp) |
| --- | --- | --- | --- |
| *Spi1* proximal for probe | 5’-GCACACATGCTTCCTGTGGTGACT-3’ | 5’-Biotin-ACCCACTGTCTAGATCGTAAGTCTA-3’ | 178 |

(D) Sequences of forward (F) and reverse (R) primers used to create probe used for EMSA.
